# Apart From the Diet, the Ruminal Microbiota of Lambs Is Modified in Relation to Their Genetic Potential for Feed Efficiency or Feeding Behavior

**DOI:** 10.3389/fmicb.2021.759432

**Published:** 2021-10-25

**Authors:** Christel Marie-Etancelin, Flavie Tortereau, Beatrice Gabinaud, Guillermo Martinez Boggio, Quentin Le Graverand, Didier Marcon, Marie-Lea De Almeida, Géraldine Pascal, Jean-Louis Weisbecker, Annabelle Meynadier

**Affiliations:** ^1^GenPhySE, INRAE, INPT, ENVT, Université de Toulouse, Toulouse, France; ^2^P3R, INRAE, Osmoy, France

**Keywords:** ruminal microbiota, feed efficiency, feeding behavior, lamb, selection

## Abstract

Using two successive types of diets (100% concentrate and 67% forage), this study explores the relationship between the ruminal microbiota of 78 Romane lambs and their feed efficiency (residual feed intake trait) or feeding behavior (feeding rate trait). Analysis was carried out phenotypically by correlating feed efficiency or feeding behavior traits with the relative abundance of bacteria at the phylum, family, and genus levels, and then genetically by comparing the microbiota of lambs selected for extreme breeding values for residual feed intake or feeding rate. Our results confirmed the major effect of diet on the ruminal microbiota composition. The microbiota of lambs consuming a forage-based diet was distinguished by higher microbial diversity and also by higher relative abundance of *Firmicutes*, whereas *Bacteriodetes* and *Actinobacteria* were relatively more abundant in the microbiota of lambs consuming a concentrate-based diet. Moreover, the comparison of lambs divergent for residual feed intake breeding values revealed that regardless of diet, more efficient lambs possessed a ruminal microbiota enriched in *Coprococcus, Moryella, [Eubacterium] Brachy group*, and *[Eubacterium] hallii group*, but depleted in *Lachnospiraceae FD2005* and *Shuttleworthia*. The connection between microbiota composition and feeding rate was more tenuous, with no link between the abundance of particular genera and lambs genetically divergent for feeding rate.

## Introduction

Feed efficiency is one of the most important phenotypic traits for animal farmers. Residual feed intake (RFI) is the most commonly used method to estimate feed efficiency, defined as the difference between the true and estimated feed intake of animals according to their body weight (BW), average daily gain (ADG), and body composition. A high RFI value characterizes low-efficiency animals since they eat more than the average population, whereas a low RFI value characterizes highly efficient animals. Selecting animals for feed efficiency is a strong lever for agroecological breeding. Individual variations in RFI can be due to a variety of factors, including ruminal digestion and the ruminal microbiome (Løvendahl et al., 2018). Indeed, the latter plays a central role in the nutrition of its host from a digestive and metabolic point of view, which directly affects feed efficiency. In particular, ruminal bacteria produce volatile fatty acids from plant-derived carbohydrates, representing approximately 70% of the animal’s energy needs (Flint et al., 2008). Beyond selection, the ruminal microbiota also provides an opportunity to control feed efficiency through dietary manipulation (Malmuthuge and Guan, 2017), which can be easily applied by farmers. Consequently, understanding host-ruminal microbiota interactions is crucial for improving farming efficiency.

Studies have reported a link between ruminal bacteria and RFI in beef cattle (Carberry et al., 2012; Hernandez-Sanabria et al., 2012; Li and Guan, 2017), dairy cows (Rius et al., 2012; Jami et al., 2014; Jewell et al., 2015), and more recently in sheep (Ellison et al., 2017; Perea et al., 2017). Among these studies, the microbiota of high-RFI (low-efficiency) animals presented increased abundance of some fibrolytic (such as *Ruminoccocus*, *Butyrivibrio*, or *Fibrobacter*) and lactolytic (such as *Anaerovibrio* or *Dialister*) genera compared to that of low-RFI animals. Moreover, the effect of RFI on the microbiota was greatly dependent on diet (Carberry et al., 2012; Hernandez-Sanabria et al., 2012; Ellison et al., 2017), although the first two studies used a non-exhaustive approach with gel electrophoresis (DGGE) to analyze the microbiota and the last study compared two groups of lambs that received different diets based on alfalfa pellets or corn. All three studies allocated conventional non-selected animals to their phenotypic RFI; however, the effect of diet was not measured in the same animals.

The feeding behavior of ruminants has been much less explored than RFI in relation to the ruminal microbiota. Breton et al. (2016) demonstrated that multiplication of *Escherichia coli* in mouse intestinal microbiota modified the animal’s satiety and feeding behavior, leading to anorexia or bulimia. Therefore, it would be interesting to examine the relationship between feeding behavior (especially feeding rate, FR) and ruminal microbiota composition. The development of automatic feeders to record feed intake and feeding duration at the visit level has enabled the analysis of feeding behavior traits. Interestingly, the FR of Romane male lambs, defined as the ratio between feed intake and feeding duration, is a heritable trait (*h*^2^ = 0.37 ± 0.06) that seems to be genetically independent of RFI (Rg = 0.08 ± 0.16) (Marie-Etancelin et al., 2019). It is thus possible to study the impact of feeding behavior and feed efficiency on ruminal microbiota without confusion between both traits.

The aim of this study was to analyze the effect of genetic selection for RFI or FR on the ruminal microbiota of Romane lambs fed two different diets, rich either in concentrate or forage.

## Materials and Methods

### Animals and Diets

**FIGURE 1:**
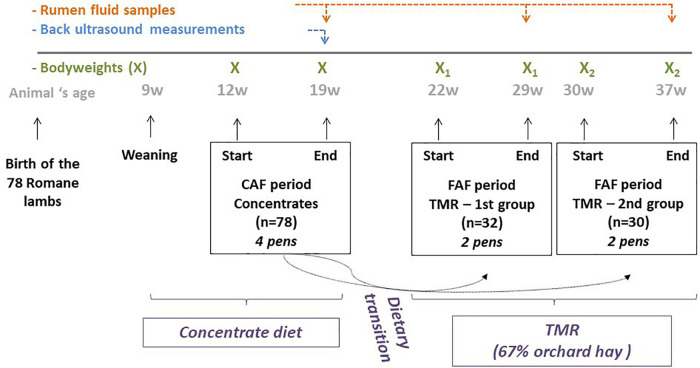
Experimental design.

A total of 78 Romane male lambs, bred at the INRAE Experimental Unit of La Sapinière (UE 332 agreement D18-174-01; Osmoy, France) in 2016, were used in this study. The lambs were reared indoors on deep straw. After weaning at 9 weeks of age, the lambs weighed an average of 26.2 kg and were adapted to a 100% concentrate diet. The experiment began at 12 weeks of age and lasted until 37 weeks of age (Figure 1). During these 26 weeks, the lambs were fed *ad libitum* by automatic feeders, which recorded individual feed intake and feeding duration at each visit. Each animal underwent two 8-week periods of feeding recording: the first period when lambs were fed concentrate using a concentrate automatic feeder (CAF period) and a second period when lambs were fed total mixed ration (TMR) using a forage automatic feeder (FAF period). The nutritional composition of the commercial concentrates used during both feeding periods is listed in Table 1. The TMR was composed of 67% orchard hay supplemented with FAF concentrate. During the first 2 weeks of each recording period, the data was not used while the lambs were adapted to the automatic feeders. At 12 weeks of age, the lambs began the 8-week CAF period, followed by 2 weeks of dietary transition to the hay diet, and then the 8-week FAF period. Due to limited availability of FAF equipment, only 62 lambs were assessed in the FAF period, which were separated into two groups: 32 lambs were recorded from 22 to 30 weeks of age during the summer and 30 lambs were recorded from 30 to 38 weeks of age during the autumn.

**TABLE 1:** Nutritional composition (/kg) of commercial concentrates used during the CAF period (100% concentrates diet) and the FAF period (66% orchard hay diet).

	**CAF concentrate**	**FAF concentrate**
Crude proteins, g/kg	157	170
Crude fat, g/kg	29	38
Crude fiber, g/kg	120	87
Starch, g/kg	151	210
Net energy, MJ/kg	6.40	6.25
Calcium, mg/kg	13	15
Phosphorus, mg/kg	4	5.8
Sodium, mg/kg	8.8	2.9
Vitamin A, UI/kg	8000	8000
Vitamin D3, UI/kg	2000	2000
Vitamin E, mg/kg	25	25
		

### Animal Measurements and Sampling

The lambs were weighed four times to obtain a 2-day average BW at the beginning and end of the 8 weeks of recording (Figure 1) for both the CAF and FAF periods. The ADG for each period was calculated from the end (eBW) and beginning BWs. Daily feed intake values for the CAF and FAF periods were averaged over each 8-week testing period to estimate the average daily feed intake (ADFI) with each diet. Muscle depth (MB) and backfat thickness (BFT) were appreciated at the end of the CAF period only, using back ultrasound measurements. The RFI was calculated as the residual value of the multiple linear regression of ADFI, ADG, metabolic BW (eBW^0^.^75^), BFT, and MD quantified at the end of the CAF period (Tortereau et al., 2020). Efficient lambs had negative RFI values.

On the last day of the CAF and FAF periods, the ruminal content was sampled for each lamb using a vacuum pump and medical gastric tube. Immobilization of the lamb was performed using a special cage adapted to lambs, and sampling was performed by competent staff. Lambs were trained to accept immobilization during animal husbandry practices. The protocol was approved by the appropriate ethical committee (APAFIS#6076-20 16070809188901).

The feed intake and feeding duration of each individual lamb were recorded for every visit during the CAF and FAF periods. For each period, the feeding behavior traits were calculated using the visit data from the last 10 days, to align as closely as possible with the rumen fluid sampling. Feeding behavior traits were defined per visit and per day. At the visit level, we analyzed the feed intake (FI/V), feeding duration (FD/V), and FR, defined as the ratio between FI/V and FD/V. At the day level, the feed intake (FI/D) and feeding duration (FD/D) were summed over 24 h, and the number of visits per day was deduced (NV/D) (Marie-Etancelin et al., 2019).

### Ruminal Bacteria Analysis

Total DNA from 85 μL ruminal fluid sample was extracted and purified using the QIAamp DNA Stool Mini Kit (Qiagen Ltd., West Sussex, United Kingdom) according to the manufacturer’s instructions with a bead-beating step using a FastPrep Instrument (MP Biomedicals, Illkirch-Graffenstaden, France). The V3–V4 region of the 16S rRNA gene was amplified from the purified genomic DNA using forward F343 (5′–CTTTCCCTACACGACGCTCTTCCGATCTACGGRAGGCAG CAG–3′) and reverse R784 (5-GGAGTTCAGACGTGTGCT CTTCCGATCTTACCAGGGTATCTAATCCT–3) primers, and the PCR was performed as described by Zened et al. (2013). Using Illumina MiSeq technology (Illumina, San Diego, CA, United States), we obtained overlapping paired-end 250-bp reads to generate full-length reads of the entire V3 and V4 regions in a single run. Single multiplexing was performed using a 6 bp index, which was added to R784 during a second PCR with 12 cycles, using forward (AATGATACGGCGACCACCGAGATCTACACTCTTTCCCTA CACGAC) and reverse (CAAGCAGAAGACGGCATACGAGAT GTGACTGGAGTTCAGACGTGT) primers. The resulting PCR products were purified, loaded onto an Illumina MiSeq cartridge (Illumina), and analyzed at the Genomic and Transcriptomic Platform (Get-PlaGe, INRAE, Toulouse, France). The raw reads were treated according to the FROGS pipeline (Escudié et al., 2018) as follows: (i) demultiplexing reads, i.e., each pair of reads was assigned to its sample with the help of the previously integrated index; (ii) preprocessing reads, i.e., merging paired-end reads using Vsearch, and removing sequences that did not match the proximal PCR primer sequences, retaining sequences with >397 nucleotides and <432 nucleotides, and removing sequences with at least one ambiguous base; (iii) clustering with the iterative Swarm algorithm executed twice and a local threshold: first with the merged reads and a maximum number of differences between sequences in each step *d* = 1, and then, with the previous cluster seed*s* and *d* = 3; (iv) removing chimeric sequences; (v) filtering operational taxonomic units (OTUs), i.e., only clusters with an abundance > 0.005% of total sequences and present in ≥6 samples were retained; and (vi) aligning sequences using the SILVA database (v. 138) (Quast et al., 2013) via BLAST. OTU affiliations were finally obtained at 3 taxonomic levels: the phylum, family, and genus levels.

### Calculations and Statistical Analysis

Bacterial alpha-diversity was estimated by the total number of observed OTUs (richness) and Shannon and Inverse-Simpson indices using the *Phyloseq* package in R software (version 3.4.1) (McMurdie and Holmes, 2013). As applied by Martinez Boggio et al. (2021) in dairy ewes, the relative abundance of taxa at the phylum, family, and genus levels was transformed by centered log ratio (CLR) using the *Compositions* package in R, after imputation of the zero values using the geometric Bayesian method (GBM). The GBM imputation was performed separately for the CAF and FAF periods in order to consider great differences in OTU distribution between the two periods.

#### Phenotypic Approach

The GLM procedure of the *sasLM* package in R software (version 3.4.1) was performed to determine the significant fixed effects affecting each transformed taxon abundance, alpha-diversity index, feeding behavior, and feed efficiency parameter. We retained the diet/feeding period effect (two levels: CAF *vs.* FAF) and the pen within feeding period effects (four levels within each feeding period) (model 1). We assumed that the feeding period effect was confounded by the diet effect. The effects of lamb “litter size,” “age” of the lamb at the beginning of the test, and “sampling order” for rumen fluid were also tested. Due to the large number of statistical tests for taxon abundance, raw *P*-values were corrected using the Benjamini–Hochberg (BH) false-discovery rate correction (Benjamini and Hochberg, 1995) leading to Q-values. Pearson correlations were estimated using residual values from the GLM analysis (model 1) between microbiota traits (relative abundance at the three taxonomic levels and alpha-diversity) and RFI during the CAF period only, or feeding behavior (FR) traits during both periods.

#### Extreme Breeding Value Approach

We selected lambs based on their extreme estimated breeding values (eBVs) for RFI or FR. Since the 78 lambs in the current study belonged to a cohort of 951 lambs reported in the genetic study by Tortereau et al. (2020), we determined eBVs for RFI based on this study. Marie-Etancelin et al. (2019) reported the feeding behavior traits of the same cohort of 951 lambs, including FR. Therefore, we calculated eBVs for FR for these 951 lambs using Pest software (Groeneveld, 1990), with a pedigree of 5,216 individuals. We then focused on the 62 lambs measured in both the CAF and FAF periods. The GLM procedure of the *sasLM* package in R was performed separately for the CAF and FAF periods to transformed taxon abundance and alpha-diversity index, considering the pen effect (4 levels) for the CAF period, and the additional seasonal effect (summer or autumn) for the FAF period (model 2). Twenty divergent lambs with extreme RFI eBVs (10 high and 10 low RFI eBVs) were compared. Additionally, twenty divergent lambs with extreme eBVs for FR (10 high and 10 low FR eBVs) were compared. The effects of the RFI/FR index groups were evaluated on the residual values of taxon abundance, alpha-diversity index, feeding behavior, and feed efficiency parameters using the GLM procedure of the *sasLM* package in R software. In order to select a small number of relevant OTUs to discriminate the RFI or FR index groups, sparse partial least square discriminant analysis (sPLS-DA) was performed using the *mixOmics* package in R software (Rohart et al., 2017) on genera residual values (model 2) separately for the CAF and FAF periods. The leave-one-out cross-validation strategy was implemented to assess the optimal numbers of components and variables per component according to the mixOmics *tuning* function, and to estimate the final model performance using the *perf* function, according to the balanced error rate (BER).

## Results

### Impact of Feeding Period

#### Animals

Descriptive statistics on the performance and feeding behavior traits of the lambs are listed in Table 2. On average, the lambs ingested 1,971 ± 272 g concentrate and 1,656 ± 339 g TMR during the CAF and FAF periods, respectively. Additionally, the lambs grew an average of 402 ± 64 g/d and 113 ± 140 g/d to reach an average BW of 52.6 ± 6.5 kg and 58.6 ± 13.6 kg at the end of the CAP and FAF periods, respectively. These values differed significantly (*P* < 0.001) between feeding periods, but reflected mixed effects of diet, season, and animal age. At the end of the CAF period, the body composition of the lambs was evaluated by dorsal ultrasound to reveal an average BFT of 7.3 ± 0.9 mm and MD of 2.73 ± 0.18 cm.

**TABLE 2:** Performance and feeding behavior traits of lambs according to the diet/period test.

		**CAF period (*N* = 78)**		**FAF period (*N* = 62)**		
		**Mean**	***SD***	**Mean**	***SD***	***P*-value**
	**Performance traits**					

	ADFI (g)	1,971	272	1,656	339	^***^
	ADG (g/j)	402	64	113	140	^***^
	eBW (kg)	52.6	6.5	58.6	13.6	^***^
	BFT (mm)	7.29	0.90	Not measured		–
	MD (cm)	2.73	0.18	Not measured		–
	RFI	−4.70	126.56	Not measured		–

	**Feeding behavior traits**					

At visit level	FI/V (g)	161	47	148	73	NS
	FD/V (min)	4.1	1.5	16.0	8.3	^***^
	FR (g/min)	41.1	12.2	9.6	2.1	^***^
At day level	FI/D (g)	2,128	351	1,693	343	^***^
	FD/D (min)	54.9	14.2	181.0	37.2	^***^
	NV/D	14.4	5.1	15.2	9.8	NS

*78 lambs received during a first period a 100% concentrates diet (CAF) and then in a second period 62 of them were kept to a 67% hay diet (FAF).ADFI, average daily feed intake; ADG, average daily gain; eBW, bodyweight at the end; BFT, backfat thickness; MD, muscle depth; RFI, residual feed intake; FI/V and FI/D, feed intake at visit and day levels, respectively; FD/V and FD/D, feeding duration at visit and day levels, respectively; FR, feeding rate; NV/D, number of visits per day.*

***P < 0.001; NS, non-significant.

With regard to feeding behavior (Table 2), the NV/D (estimated over the last 10 days of each period) did not differ significantly between feeding periods, with average NV/D values of 14.4 ± 5.1 and 15.2 ± 9.8 for the CAF and FAF periods, respectively. The average FI/V value seemed to be higher in the CAF period (161 ± 47 g) than in the FAF period (148 ± 73 g), although this difference was not significant. Moreover, the feeding duration was over 3 times longer during the FAF period than during the CAF period, both for FD/V (16.0 ± 8.3 min *vs.* 4.1 ± 1.5 min) and FD/D (181.0 ± 37.2 min *vs.* 54.9 ± 14.2 min). This difference in duration led to significantly (*P* < 0.001) dissimilar FRs between the two feeding periods, with average FR values of 41.1 ± 12.2 g/min and 9.6 ± 2.1 g/min for the CAF and FAF periods, respectively. Regardless of the trait, the effects of “litter size,” “age” of the lamb at the beginning of the test, and “sampling order” were not significant.

#### Microbiota

For the 140 samples, 2,808,537 sequences were produced by the Illumina MiSeq technology. After the pre-process step, 2,520,572 sequences were retained and grouped into 336,894 clusters, using Swarm algorithm. The deletion of chimeric sequences and the filtering of clusters with an abundance lower than 0.005% of the total sequences, leads to the deletion of 216,357 and 362,771 sequences corresponding to 122,543 and 212,852 clusters, respectively. Thus, without a rarefying step, we obtained 1,499 OTUs corresponding to 1,941,444 sequences for 140 samples (78 CAF period samples and 62 FAF period samples) that is to say 13,800 sequences per sample on average. 99.5, 99.0, and 98.0% of the 1,499 OTUs were successfully affiliated at the phylum, family, and genus levels, respectively; the 100% complement corresponded to multi-affiliations. Because the affiliation rate dropped <8% at the species level, we decided not to explore this taxonomic rank. Finally, the microbial community was structured into 114 different bacterial genera, which were grouped into 54 families and 10 phyla.

**FIGURE 2:**
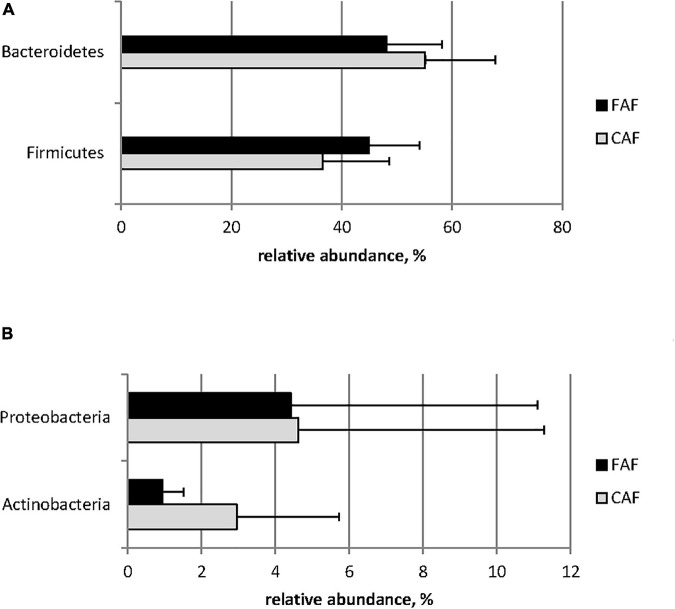
Means of major **(A)** and minor **(B)** phylum (relative abundance > 1%) significantly (*Q* < 0.001) affected by the diet/period test: 78 lambs received during a first period a 100% concentrates diet (CAF, 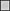) and then in a second period 62 of them were kept to a 67% hay diet (FAF, 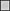).

The relative abundance of phyla was greatly affected by diet (Figure 2). *Firmicutes* was significantly more abundant in the FAF period compared to the CAF period, whereas *Bacteroidetes* was significantly less abundant in the FAF period (Figure 2A; *Q* < 0.001). The relative abundance of *Actinobacteria* and *Proteobacteria* was significantly decreased when lambs were switched from the concentrate-based to the forage-based diet (CAF to FAF period) (Figure 2B; *Q* < 0.001). Among other minor phyla (relative abundance ranging from 0.1 to 0.7% of total sequences), *Desulfobacterota* and *Fibrobacterota* were slightly affected by diet (*Q* < 0.05), with increased abundance in the FAF period compared to the CAF period, while *Spirochaetes* was not affected. The presence of *Fusobacteria* was specific to the FAF period (i.e., undetected during the CAF period).

**FIGURE 3:**
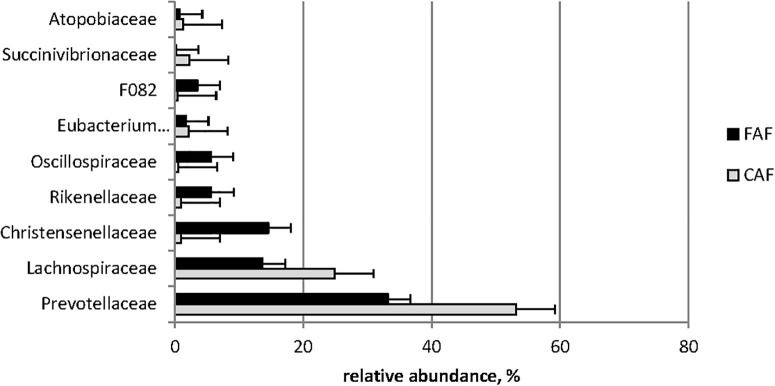
Means of ruminal dominant families (relative abundance > 1%) significantly (*Q* < 0.001) affected by the diet: 78 lambs received during a first period a 100% concentrates diet (CAF, 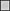) and then in a second period 62 of them were kept to a 67% hay diet (FAF, 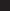).

Based on the BH-corrected P-values, 41/54 bacterial families and 91/114 bacterial genera were affected by diet (*Q* < 0.05). The switch from the concentrate-based to the forage-based diet resulted in a sharp reduction in the abundance of *Lachnospiraceae* (25% to 14%) and *Prevotellaceae* (53–33%), and a strong increase in *Christensenellaceae* (1–15%) and *Rikenellaceae* (1–6%) abundance families (Figure 3).

**FIGURE 4:**
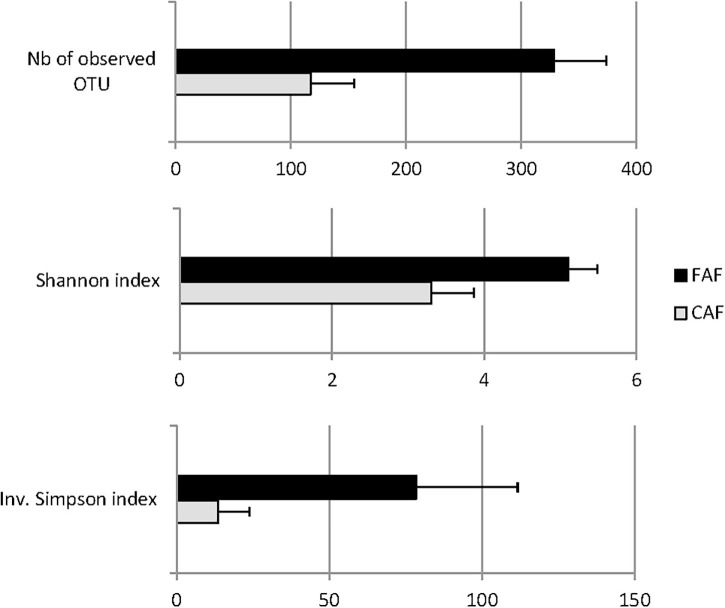
Least-squares means of alpha-diversity traits of lambs’ rumen microbiota significantly different (*P* < 0.001) according to the diet: 78 lambs received during a first period a 100% concentrates diet (CAF, 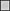) and then in a second period 62 of them were kept to a 67% hay diet (FAF, 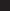).

The results indicated that microbiota diversity was strongly affected by diet (Figure 4). Feeding lambs with forage significantly (*P* < 0.001) increased the diversity of the ruminal microbiota considering the evenness of species distribution. Indeed, the Shannon and Inverse Simpson indexes increased from 3.33 to 5.11 and from 14.0 to 78.5 from the CAF to FAF periods, respectively, and the number of observed OTUs increased from 119 to 329.

### Feed Efficiency and Microbiota Composition

#### Phenotypic Approach

Phenotypic correlations with RFI could only be estimated during the CAF period, since RFI was not calculated during the FAF period. RFI was not significantly correlated with microbiota alpha-diversity traits nor abundance, regardless of taxonomic level.

#### Extreme Breeding Value Approach

The average difference in eBVs between the two RFI index groups was 146.5 g/d, which is approximately 2 genetic standard deviations (Table 3) since the genetic standard deviation of RFI was estimated at 73 g/d (Tortereau, 2020; personal communication).

**TABLE 3:** Breeding values of extreme animals for residual feed intake or feeding rate.

	**Index**				**Differences between**
	**Mean**	**SD**	**Min**	**Max**	**BV groups**
**RFI-BV (g/d)**					

10 RFI-BV− (efficient)	–72.4	25.8	–46.6	–121.2	146.5 g/d
10 RFI-BV+ (inefficient)	+ 74.1	12.3	+ 92.8	+ 58.0	i.e., ∼2 sd

**FR-BV (g/min)**					

10 FR-BV− (slow)	–7.53	0.65	–8.76	–6.57	10.90 g/min
10 FR-BV+ (rapid)	+ 3.37	1.65	+ 1.65	+ 6.51	

*RFI-BV, residual feed intake breeding values; FR-BV, feeding rate breeding values.*

The performance traits of the 20 lambs with extreme RFI eBVs during the CAF period are presented in Table 4. As expected, the two RFI index groups differed significantly (*P* < 0.001) for RFI (difference of 266.6 g/d), ADFI (difference of 309 g/d), and FI/D (difference of 281 g/d). No other traits were differentiated with phenotypic RFI, neither feed efficiency nor feeding behavior traits. Additionally, the RFI index groups did not differ in terms of their microbiota alpha-diversity traits nor their relative abundance during the CAF and FAF periods, regardless of taxonomic level.

**TABLE 4:** Performance and feeding behavior traits of lambs for extreme RFI index and FR index during 100% concentrates CAF period.

		**RFI-BV− (efficient)**	**RFI-BV+ (inefficient)**			**FR-BV+ (rapid)**	**FR-BV− (slow)**		
		**Lsmeans**	**Lsmeans**	***s.e.***	***P*-value**	**Lsmeans**	**Lsmeans**	***s.e*.**	***P*-value**
**Feed efficiency traits**									

	ADFI (g)	**1,748**	**2,057**	**61**	^**^	2,073	1,939	68	NS
	ADG (g/j)	385	395	17	NS	407	406	15	NS
	eBW (kg)	50.6	51.5	1.9	NS	54.0	53.0	1.4	NS
	BFT (mm)	6.97	7.42	0.30	NS	7.64	6.96	0.29	NS
	MD (cm)	2.72	2.70	0.07	NS	2.70	2.77	0.04	NS
	RFI	**−135.7**	**+130.9**	**26.7**	^***^	−67.0	+29.9	43.6	NS

**Feeding behavior traits**									

At visit level	FI/V (g)	147	137	13	NS	170	159	13	NS
	FD/V (min)	4.0	3.1	0.3	NS	**3.1**	**4.7**	**0.3**	^***^
	FR (g/min)	38.6	44.6	3.5	NS	**55.1**	**33.7**	**2.1**	^***^
At day level	FI/D (g)	**1.916**	**2.197**	**91**	^*^	2.260	2.085	104	NS
	FD/D (min)	51.7	51.7	3.9	NS	**41.1**	**63.2**	**3.1**	^***^
	NV/D	13.4	17.9	1.6	NS	13.9	13.7	1.0	NS

*RFI-BV, residual feed intake breeding values; FR-BV, feeding rate breeding values; ADFI, average daily feed intake; ADG, average daily gain; eBW, bodyweight at the end; BFT, backfat thickness; MD, muscular fat; FI/V and FI/D, feed intake at visit and day levels, respectively; FD/V and FD/D, feeding duration at visit and day levels, respectively; FR, feeding rate; NV/D, number of visits per day.*

***P < 0.001, **P < 0.01, *P < 0.05; NS, non-significant; in bold, significant differences.

**FIGURE 5:**
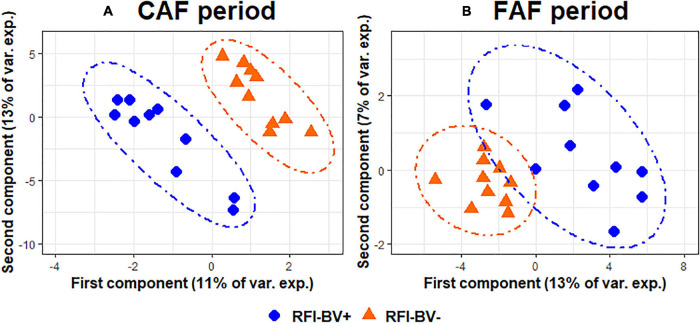
Sparse partial least squares discriminant analysis of ruminal bacteria abundances at the genus level according to Residual Feed Intake index groups (*N* = 20) during 100% concentrate CAF (**A**- sPLS-DA with 3 components, 8, 50, and 90 variables per component and a BER of 0.20) and 67% forage FAF (**B**- sPLS-DA with 3 components, 75, 1, and 1 variables per component and a BER of 0.10) periods.

The sPLS-DA on the GLM residual values of taxa abundance at the genus level enabled discrimination of the RFI index groups (Figure 5A) by selecting three components with 8, 50, and 90 variables per component in the CAF period. The BER of this model was 0.20. On the first axis, the selected genera (Table 5) all belonged to the phylum *Firmicutes*, with the most efficient lambs (RFI-BV−) having increased abundance of two genera (*Coprococcus* and *Lachnospiraceae Probable genus 1*) and decreased abundance in three genera (*Colidextribacter*, *Lachnospiraceae ND3007*, and *Selenomonas*) compared to less efficient lambs. Different phyla were represented on the second axis, with increased abundance of the genera *Atopobium*, *Moryella*, *[Eubacterium] brachy group*, and *[Eubacterium] hallii group* in efficient lambs, whereas inefficient lambs had increased abundance in the genera *Oscillospiraceae UCG-007*, *Ruminococcaceae UCG-001*, *Streptococcus*, *Candidatus soleaferrea*, and *[Eubacterium] xylanophilum group*. For the same lambs during the FAF period, the sPLS-DA on the GLM residual values of taxa abundance at the genus level enabled discrimination of the RFI index groups (Figure 5B) by selecting three components with 75, 1, and 1 variable per component. The BER was 0.10, indicating good prediction ability. On the first axis, the selected taxa were numerous (*n* = 75). Among those with the highest loading values (Table 5), some genera were associated with more efficient lambs (*Sediminispirochaeta*, *[Eubacterium] brachy group*, and *Moryella*), while some were associated with inefficient lambs (*Undibacterium*, *Lachnospiraceae FD2005*, and *Prevotellaceae UCG-003*). On the second axis, only the genus *Oribacterium* was selected and associated with efficient lambs. The 3rd axis of the analysis discriminated very little between the RFI index groups during either the CAF or FAF periods.

**TABLE 5:**
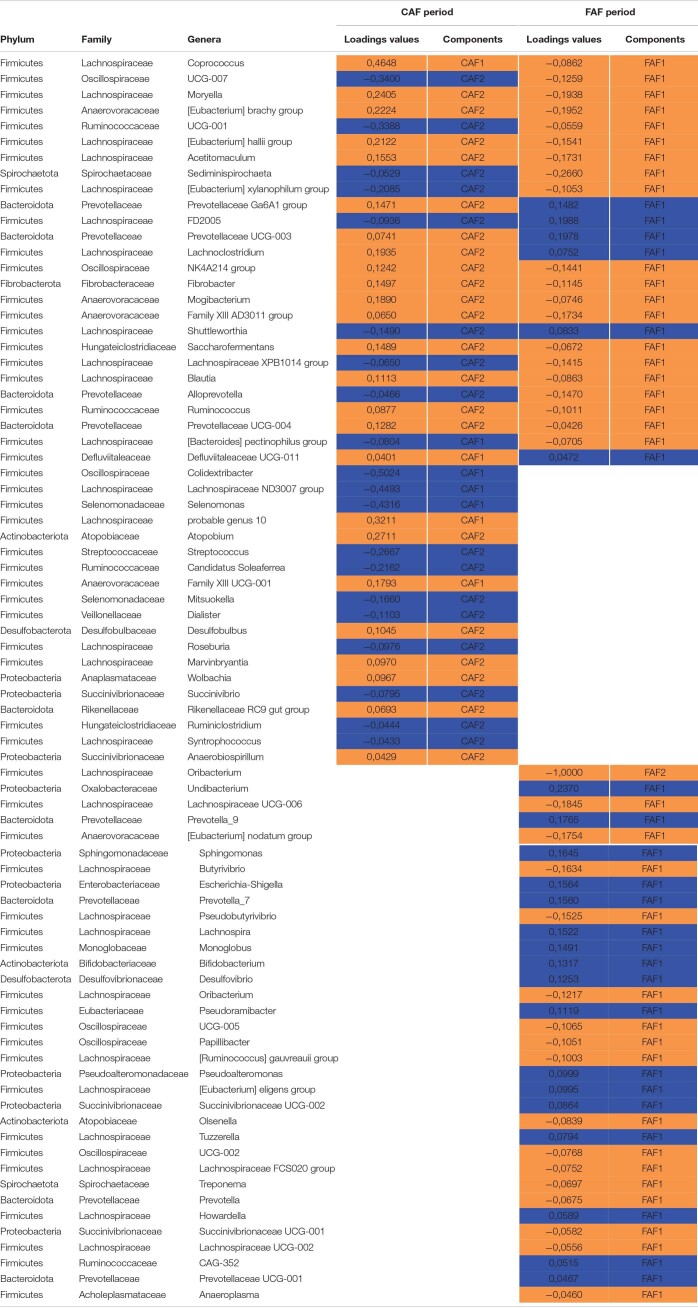
Sparse partial least squares discriminant analysis of ruminal bacteria abundances at the genus level according to residual feed intake index groups (*N* = 20) during 100% concentrate CAF (a) and 67% forage FAF (b) periods.

*Variables and loadings selected on the 2 first components (CAF1, CAF2 for first and second components during CAF period and FAF1, FAF2 for first and second components during FAF period), in orange and blue genera associated with RFI-BV− and RFI-BV+, respectively.*

Finally, comparing genera selected in each feeding periods to discriminate the RFI index groups, 19 and 34 genera were specific to the CAF and FAF periods, respectively, while 26 genera were common to both feeding periods (Table 5). Among them, the abundance of 15/26 genera varied in the same manner in the CAF and FAF periods: *Coprococcus*, *Moryella*, *[Eubacterium] brachy group*, *[Eubacterium] hallii group*, *Acetitomaculum*, *Oscillospiraceae NK4A214*, *Fibrobacter*, *Mogibacterium*, *Family XIII AD3011*, *Saccharofermentass*, *Blautia*, *Ruminococcus*, and *Prevotellaceae UCG-004* displayed higher abundance in the RFI-BV− group, while *Lachnospiraceae FD2005* and *Shuttleworthia* displayed higher abundance in the RFI-BV+ group. The relative abundance of the remaining 11/26 genera varied in opposite directions in RFI index groups in the CAF and FAF periods (*Oscillospiraceae UCG-007*, *Ruminococcaceae UCG-001*, *Sediminispirochaeta*, *[Eubacterium] xylanophilum group*, *Prevotellaceae Ga6A1*, *Prevotellaceae UCG-001*, *Lachnoclostridium*, *Lachnospiraceae XPB1014*, *Alloprevotella*, *[Bacteroides] pectinophilus group*, and *Defluviitaleaceae UCG-001*).

### Feeding Rate and Microbiota Composition

#### Phenotypic Approach

Feeding rate was not significantly correlated with microbiota alpha-diversity traits nor abundance at the phylum, family, or genus levels during the CAF and FAF periods.

#### Extreme Breeding Value Approach

The average difference in eBVs between the two FR index groups was 10.90 g/min (Table 3). As this trait was not evaluated in the whole Romane lamb population, it was not possible to transpose this difference into an equivalent genetic standard deviation.

The performance and feeding behavior traits of lambs with extreme FR eBVs are presented in Table 4. As expected, the average FR values differed significantly between the two FR index groups with a ratio of 1.6 (33.7 g/min *vs.* 55.1 g/min; *P* < 0.001). Lambs in the FR index groups also significantly diverged for FD/V (3.1 min vs. 4.7 min; P < 0.01) and FD/D (41.1 min vs. 63.2 min; *P* < 0.001), the highest FR the shortest feeding duration. NV/D values did not differ significantly between the FR index groups. The FR index groups had also similar feed efficiency traits, including RFI. Comparing the ruminal microbiota during the CAF and FAF periods, the FR index groups did not differ in terms of their microbiota alpha-diversity traits nor their relative abundance, regardless of taxonomic level.

**FIGURE 6:**
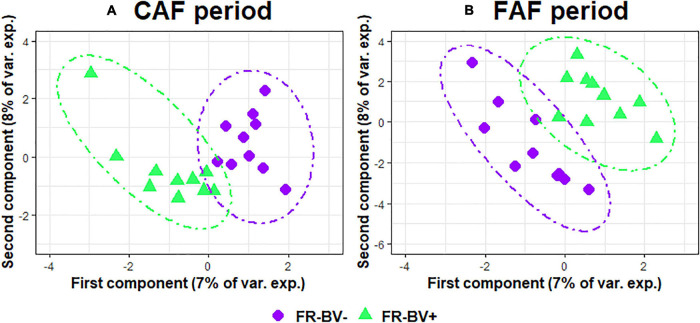
Sparse partial least squares discriminant analysis of ruminal bacteria abundances at the genus level according to feeding rate index groups (*N* = 20) during 100% concentrate CAF (**A**- sPLS-DA with 2 components, 4 and 6 variables per component and a BER of 0.40) and 67% forage FAF (**B**- sPLS-DA with 1 component and 3 variables, and a BER of 0.45) periods.

The two sPLS-DA on the GLM residual values of taxa abundance at the genus level enabled very little discrimination of the FR index groups for the CAF and FAF periods (Figures 6A,B), since the BERs were 0.40 and 0.45, respectively. During the CAF period, four and six taxa on the first and second axes were selected, respectively (Table 6). Among them, the relative abundance of genera *Clostridium sensu stricto 1*, *Lachnospiraceae NK3A20*, and *Colidextribacter* increased in the FR-BV+ group, whereas the relative abundance of genera *[Eubacterium] eligens group*, *Lachnospiraceae AC2044*, and *Family XIII AD3011* increased in the FR-BV− group. Only the first axis was used to discriminate the FR index groups during the FAF period, and three genera were retained. The relative abundance of *Prevotellaceae UCG-001* increased in the FR-BV− group, whereas that of *Lachnospiraceae AC2044* and *Prevotellaceae UCG-001* increased in the FR-BV+ group. Among the 11 selected genera, only eight were discriminant in the CAF period, one was specific to the FAF period, and two genera were discriminant in both periods (*Lachnospiraceae AC2044* and *Prevotellaceae UCG-001*), but their abundance varied in opposite manner between FR index groups (Table 6).

**TABLE 6:**
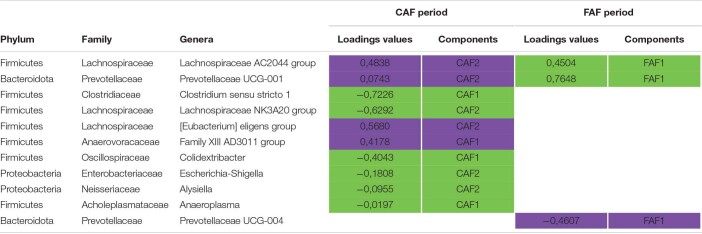
Sparse partial least squares discriminant analysis of ruminal bacteria abundances at the genus level according to Feeding Rate index groups (*N* = 20) during 100% concentrate CAF (a) and 67% forage FAF (b) periods.

*Variables and loadings selected on the 2 first components (CAF1, CAF2 for first and second components during CAF period and FAF1, FAF2 for first and second components during FAF period), in green and purple genera associated with FR-BV+ and FR-BV−, respectively.*

## Discussion

### The Major Effect of Diet

In this study, feeding behavior was affected by diet. During the CAF period, lambs ate more and faster than during the FAF period. Such behavior in lambs has been previously reported. For example, Sari et al. (2018) reported increased FI/V and FR with increasing concentrate level in the diet, which may have been due to improved palatability and higher rates of ruminal degradation and passage of concentrates compared to forage. Moreover, the ruminal microbiota is responsible for ruminal degradation of food, which might play a role in the observed differences in eating behavior.

Our results confirmed the major impact of the type of food ingested by the lambs on the composition of their microbiota (Ellison et al., 2017). The three alpha-diversity traits were greatly increased when lambs ingested forage rather than concentrate. Depending on the trait, the alpha-diversity was multiplied a factor of 1.5 to 5. The lower bacterial diversity in the rumen of cattle fed high levels of concentrate compared to high levels of forage has been well established (Mao et al., 2013; Zened et al., 2013; McGovern et al., 2020). Ellison et al. (2014) previously reported substantial changes to sheep microbiota consuming concentrate-based or forage-based diets, with a more diverse microbial ecosystem observed for the forage-based diet. These authors proposed two likely arguments: the ruminant microbiota is more adapted to forage than concentrate digestion, and forage-based diets are more diversified in nutrients than concentrate-based diets.

Diet affected the relative abundance of almost all bacteria, regardless of taxonomic level. Indeed, 9/10 phyla, 41/54 families, and 91/114 genera were significantly impacted. The ruminal ecosystem under the forage-based diet was characterized by increased relative abundance of *Firmicutes* and *Fibrobacteres*, whereas that of *Bacteroidetes* and *Actinobacteria* decreased compared to the concentrate-based diet. At the family level, the concentrate-based diet led to higher relative abundance of *Prevotellaceae* and *Lachnospiraceae* and lower relative abundance of *Ruminococcaceae*, *Christensenellaceae*, and *Rikenellaceae* than the forage-based diet. Some studies reported that *Bacteroidetes* (Fernando et al., 2010; Zhang et al., 2019) and *Prevotella* (Henderson et al., 2015) were more abundant in animals fed diets containing concentrate. However, studies have also reported opposite results under high-concentrate diets (Mao et al., 2013; Zened et al., 2013). The literature is also inconsistent concerning *Firmicutes*, *Lachnospiraceae*, and *Ruminococcaceae*. The relative abundance of these bacteria increased with concentrate incorporation in some studies, but other studies reported the opposite effect (Fernando et al., 2010; Mao et al., 2013; Zened et al., 2013; Zhang et al., 2019). These differences could be explained by other features of the diet and sampling conditions, such as the nature of the forage and the sampling site (Deusch et al., 2017). For other groups of bacteria, our results were in accordance with previous studies in which increasing concentrate proportions reportedly led to decreased abundance of *Rikenellaceae* (Zened et al., 2013; Zhang et al., 2019), *Christensenellaceae* (Zhang et al., 2019) and *Fibrobacteres* (Fernando et al., 2010; Zhang et al., 2019), and increased abundance of *Actinobacteria* (Mao et al., 2013; Zened et al., 2013).

### Microbiota Changes Due to Selection for Residual Feed Intake or Feeding Rate

Beyond the major effect of diet on the ruminal microbiota, we tested the impact of selection for RFI or FR. Even if differences between groups of lambs divergent for RFI or FR were significant, the impact on the diversity of the bacterial community appeared to be null. Thus, we did not highlight any link between RFI or FR index groups and microbiota alpha-diversity traits (observed OTUs, or Shannon and Inverse-Simpson indexes). Moreover, no significant phenotypic correlations were obtained between alpha-diversity traits and RFI or FR phenotypes in the whole dataset. Regarding feed efficiency, our results were consistent with those of previous studies (Li and Guan, 2017; McGovern et al., 2018, 2020; Patil et al., 2018) that reported no differences in alpha-diversity traits between low- and high-RFI steers or lambs. However, Shabat et al. (2016) reported significantly lower microbiome richness and a lower Shannon index in efficient dairy cows compared to less efficient cows, regardless of taxonomic level. Li et al. (2016) concluded that bacterial community structures differed between high- and low-RFI steers after applying a weighted UniFrac test to measure beta-diversity. Interestingly, Li and Guan (2017) used the Bray–Curtis dissimilarity test to measure beta-diversity in the same dataset, concluding there was no clear separation between groups of high- and low-RFI steers. McGovern et al. (2020) reported no difference between two groups of steers phenotypically divergent for RFI, regardless of the beta-diversity test used.

Comparing the relative abundance of taxa clearly demonstrated that diet influenced the lamb microbiome more than feed efficiency (RFI) or FR status. In our dataset, diet impacted 91/114 bacterial genera (*Q* < 0.05), whereas RFI or FR traits did not display significant links with bacterial genera. To a lesser extent, Ellison et al. (2017) previously quantified this disequilibrium between diet type and feed efficiency in 16 sheep, reporting that the abundance of 44/349 OTUs differed with respect to diet while only 11/349 differed with respect to RFI status. In bovines, McGovern et al. (2020) found no difference in microbial taxa between RFI phenotypes, and only ten OTUs were significantly associated with RFI, independent of other factors of variation such as diet, breed, and age.

In the present study, some bacterial genera were impacted by genetic RFI during both feeding periods, with a high ability of prediction (error rates of 0.20 and 0.10 during the CAF and FAF periods, respectively). Our results indicated decreased abundance of *Lachnospiraceae FD2005* and *Shuttleworthia* and increased abundance of *Coprococcus*, *Moryella, Eubacterium Brachy*, *Eubacterium hallii*, *Acetitomaculum*, *Oscillospiraceae NK4A214*, *Fibrobacter*, *Mogibacterium*, and *Family XIII AD3011* in efficient lambs compared to the inefficient lambs, regardless of diet. Notably, among the 26 genera that discriminated the RFI index groups in both the CAF and DAF periods, 11 belonged to the family *Lachnospiraceae* (*Coprococcus*, *Moryella*, *Eubacterium Hallii, Acetitomaculum*, *Eubacterium xylanophilum*, *Lachnospiraceae FD2005*, *Lachnoclostridium, Shuttleworthia, Lachnospiraceae XPB1014*, *Blautia*, and *Bacteroides pectinophilus*) and four belonged to the family *Prevotellaceae* (*Prevotellaceae Ga6A1*, *Prevotellaceae UCG-003, Prevotellaceae UCG-004*, and *Alloprevotella*), demonstrating opposite correlations with RFI within the family. Differing correlations between RFI and bacterial genera within the same family might explain the lack of significant link between RFI and these bacterial families, particularly *Lachnospiraceae*. Bovine literature often reports a positive relationship between *Lachnospiraceae* abundance and RFI, with inefficient animals having higher abundance (Rius et al., 2012; Jewell et al., 2015; Li et al., 2016; Shabat et al., 2016; Li and Guan, 2017; Perea et al., 2017; McGovern et al., 2018). McGovern et al. (2018) reported a correlation of +0.44 between RFI and *Lachnospiraceae* abundance in the solid ruminal phase only. At the genus level, some of these studies (Jewell et al., 2015; Shabat et al., 2016; McGovern et al., 2018) have also reported that some OTUs belonging to the family *Lachnospiraceae* are positively or negatively correlated with RFI, which concurred with our results. These inconsistent effects could also depend on diet, as suggested by the opposite links observed in the CAF and FAF periods for the *[Eubacterium] xylanophilum group*, *Lachnoclostridium*, *Lachnospiraceae XPB1014*, and *[Bacteroides] pectinophilus group* (Table 5). The relationship between the ruminal microbiome and some functional aspects, such as feed efficiency, needs to be further investigated at the genus or OTU level, as was conducted in the most recent studies (Auffret et al., 2020; McGovern et al., 2020; McLoughlin et al., 2020).

At the genus level, we observed increased *Coprococcus* abundance under both diets in efficient lambs. These results were consistent with the literature (Jewell et al., 2015; Shabat et al., 2016; McGovern et al., 2018), in which a negative relationship was reported between RFI and *Coprococcu*s abundance in the liquid rumen content. Moreover, Shabat et al. (2016) also reported an enrichment of *Coprococcus* in their efficient cow rumen. Additionally, our study findings indicated a higher abundance of the genus *Moryella* associated with efficient lambs, as in the study by Hernandez-Sanabria et al. (2012), which was positively linked with RFI in the study by Jami et al. (2014). Further, *Acetitomaculum* abundance was higher in efficient lambs in the current study. Considering the feed conversion ratio as a measurement of feed efficiency, McLoughlin et al. (2020) reported the same trend. This genus belongs to acetogenic bacteria (Le Van et al., 1998), which can use H_2_ to reduce CO_2_ to acetate and thus limit methane emission, leading to decreased energy loss. *Blautia* was another acetogenic bacterium with increased abundance in efficient lambs in our study, which was also reported by Nakamura et al. (2010). Moreover, we observed a negative link between *Fibrobacter* and RFI, as reported between the phylum *Fibrobacteres* (McGovern et al., 2018), the family *Fibrobacteraceae* (Rius et al., 2012), or *Fibrobacter* sp. (Perea et al., 2017) and RFI, in that efficient lambs had greater abundance of *Fibrobacteres/Fibrobacteraceae*. Although McGovern et al. (2018) reported strong correlations (between −0.43 and −0.59) in solid and liquid rumen phases between RFI and *Fibrobacteres succinogenes*, this link was not corroborated in other studies (Jewell et al., 2015; Shabat et al., 2016). Moreover, McGovern et al. (2020) found that *Mogibacterium* was negatively associated with phenotypic RFI, which concurred with the results of our study. These bacteria are saccharolytic and thought to be mainly involved in ammonia assimilation (Nakazawa et al., 2000). Rius et al. (2012) reported that efficient cattle (low RFI) had higher nitrogen digestion than inefficient cattle, suggesting a possible link between feed efficiency and ruminal nitrogen metabolism.

McGovern et al. (2018) reported that *Ruminococcaceae* and *Ruminococcus* abundance increased in animals with high feed efficiency, in particular *Ruminococcus flavefaciens*, while their abundance was reportedly increased in low-efficiency animals in the study by Jewell et al. (2015). In a study by Shabat et al. (2016), *Ruminococcus albus* and *R. flavefaciens* were associated with H_2_ (used by *Archaea* for methane production) and CO_2_ production, and so, in spite of one strain of *R. flavefaciens*, their abundance was increased in low-efficiency animals. At the genus level, we highlighted the opposite effects between *Ruminococcaceae UCG001* and *Ruminococcus* abundance and RFI when lambs consumed the concentrate-based diet, but efficient lambs had lower percentages of these two genera in their rumen when they consumed the forage-based diet.

During the FAF period only, the abundance of *Oribacterium* increased in efficient sheep compared to inefficient sheep, while McGovern et al. (2020) observed a positive link between this genus and RFI regardless of diet. *Oribacterium* is a genus strongly involved in cell wall degradation (Kang et al., 2019), contributing to one of the ruminal paradoxes. The efficiency of plant cell wall digestion driven by fibrolytic bacteria conditions access to nutrients for all bacteria and increases overall digestion efficiency in the rumen, while increasing parallel energy loss through methane emissions. Such complex phenomena could explain, at least in part, why no clear link could be established between fibrolytic bacteria and feed efficiency. *Shuttleworthia* was one of two taxa with increased abundance in inefficient lambs regardless of diet, but this result opposes that reported by Jewell et al. (2015). Given the lack of information on this genus, no hypotheses can be drawn.

Shabat et al. (2016) and Auffret et al. (2020) proposed an explanation for the relationship between feed efficiency and particular ruminal microbiota composition: butyrate and lactate producers (and in general bacteria using the acrylate pathway, i.e., *Coprococcus*) would offer more relevant output metabolites for animals than succinate and acetate/H_2_ producers (e.g., *Ruminoccocus*). Although these authors seemed to agree on the higher efficiency of acrylate metabolism compared with succinate metabolism, the butyrate hypothesis remains debated because of the difficulties correlating *Lachnospiraceae* (butyrate producer) and RFI. Moreover, in growing animals like lambs, the propionate pathway is known to be more efficient for growth than acetate and butyrate. Lactate-producing bacteria, especially prominent during the CAF period, were more abundant in both efficient (e.g., *Coprococcus* and *Atopobium*) and inefficient lambs (e.g., *Colidextribacter* and *Streptococcus*). Here again, it is difficult to establish a clear link, probably because lactate, although a metabolically efficient nutrient, may also enhance acidogenic rumen conditions, inhibit fibrolytic bacteria, and thus reduce ruminal digestion efficiency. However, the association between *Coprococcus* and low RFI is most frequently reported in the literature (Jewell et al., 2015; Shabat et al., 2016; McGovern et al., 2018), although sometimes not observed (Jami et al., 2014), and there is currently no report of increased *Coprococcus* abundance in inefficient animals. Another explanation is the ability of *Coprococcus* to ferment aromatic compounds into acetate, benzoate, and organic acids (Patel et al., 1981). Moreover, a syntrophic action exists between *Coprococcus* and *Papillibacter*, in which *Coprococcus* ferments benzoate produced by *Papillibacter* (Defnoun et al., 2003). In our study, *Papillibacter* was associated with efficient lambs, but only during the FAF period. In humans, such compounds have been shown to reduce obesity in association with changes in gut microbiota and energy metabolism (Van Hul et al., 2018). Since the overall objective is to increase weight gain in growing animals, fermentative bacteria that can utilize aromatic compounds may play a potential role in feed efficiency. Sung et al. (2017) suggested that microbiota sensitive to such compounds (such as *Moryella*) may, at least in part, act on largely undigested aromatic compounds (i.e., polyphenol) in humans. Aromatic compounds, particularly plant polyphenols, are known to be bioactive and alter ruminal digestion (Vasta et al., 2019). Therefore, the effect of aromatic compounds on rumen bacteria needs to be investigated with respect to feed efficiency.

The impact of genetic FR on microbiota taxa abundance was even smaller than that observed with genetic RFI, and to date has not been reported in the literature. The only relevant study (Schären et al., 2018) did not find a clear association between feeding behavior (volume, duration, and frequency) and rumen OTUs in dairy cows. The relative abundance of rumen bacteria in the current study did not enable discrimination of the FR index groups (error rates of 0.40 and 0.45 during the CAF and FAF periods, respectively). Moreover, none of the bacteria selected by sPLS-DA in the feeding periods were correlated in the same manner in the FR index groups. Therefore, it is difficult to conclude a link between the ruminal microbiota and selection for FR, and further investigation is warranted to advance possible explanations.

## Conclusion

Although we did not notice an impact on microbial diversity, feed efficiency was linked with some ruminal microbial genera, particularly bacteria involved in the degradation of aromatic compounds (such as *Coprococcus*), nitrogen metabolism (such as *Mogibacterium*), and acetogenesis (such as *Actetitomaculum*). The link between feed efficiency and plant cell wall digestion efficiency requires further study due to its complex relationship. Surprisingly, genetic FR was only slightly related with a specialized microbiota, which was much less than with genetic RFI. Nevertheless, as expected, diet had a major impact on the ruminal microbial community, and some associations between the microbiota and feed efficiency or feeding behavior could be dependent on diet. Our work continues with divergent RFI selection and a larger number of lambs to explore links between the presence of bacteria in the microbiota and their metabolite production, assaying ruminal and blood metabolites in association with ruminal microbiota analysis.

## Data Availability Statement

The datasets presented in this study can be found in online repositories. The names of the repository/repositories and accession number(s) can be found below: https://www.ncbi.nlm.nih.gov/, bioproject/755177.

## Ethics Statement

The animal study was reviewed and approved by the appropriate ethical committee (APAFIS#6076-20 16070809188901). Written informed consent was obtained from the owners for the participation of their animals in this study.

## Author Contributions

AM, CM-E, and FT designed and monitored the experiments. J-LW and DM implemented the experiments in the P3R unit. BG and M-LD performed the laboratory analysis of the 16S sequencing. GP performed the bioinformatics analysis of the sequences. CM-E performed the statistical analysis of the data with the help of GMB and QL. CM-E drafted the manuscript with contributions from all co-authors. All authors contributed to the article and approved the submitted version.

## Conflict of Interest

The authors declare that the research was conducted in the absence of any commercial or financial relationships that could be construed as a potential conflict of interest.

## Publisher’s Note

All claims expressed in this article are solely those of the authors and do not necessarily represent those of their affiliated organizations, or those of the publisher, the editors and the reviewers. Any product that may be evaluated in this article, or claim that may be made by its manufacturer, is not guaranteed or endorsed by the publisher.
